# Reduced cold tolerance of viral-infected leafhoppers attenuates viral persistent epidemics

**DOI:** 10.1128/mbio.03211-23

**Published:** 2024-04-02

**Authors:** Biao Chen, Gehui Cao, Yulu Chen, Tong Zhang, Guohui Zhou, Xin Yang

**Affiliations:** 1 Guangdong Province Key Laboratory of Microbial Signals and Disease Control, College of Plant Protection, South China Agricultural University, Guangzhou, China; University of California at Riverside, Riverside, California, USA

**Keywords:** rice stripe mosaic virus, *Recilia dorsalis*, cuticular protein gene, vsiRNA, cold tolerance

## Abstract

Most arthropod-borne viruses produce intermittent epidemics in infected plants. However, the underlying mechanisms of these epidemics are unclear. Here, we demonstrated that rice stripe mosaic virus (RSMV), a viral pathogen, significantly increases the mortality of its overwintering vector, the leafhopper species *Recilia dorsalis*. Cold-stress assays indicated that RSMV reduces the cold tolerance of leafhoppers, a process associated with the downregulation of leafhopper cuticular protein genes. An RSMV-derived small RNA (vsiR-t00355379) was found to facilitate the downregulation of a leafhopper endocuticle gene that is mainly expressed in the abdomen (named RdABD-5) and is conserved across dipteran species. The downregulation of *RdABD-5* expression in *R. dorsalis* resulted in fewer and thinner endocuticle lamellae, leading to decreased cold tolerance. This effect was correlated with a reduced incidence rate of RSMV in early-planted rice plants. These findings contribute to our understanding of the mechanism by which viral pathogens reduce cold tolerance in arthropod vectors and suggest an approach to managing the fluctuating prevalence of arboviruses.

**IMPORTANCE:**

Increasing arthropod vector dispersal rates have increased the susceptibility of crop to epidemic viral diseases. However, the incidence of some viral diseases fluctuates annually. In this study, we demonstrated that a rice virus reduces the cold tolerance of its leafhopper vector, *Recilia dorsalis*. This effect is linked to the virus-derived small RNA-mediated downregulation of a gene encoding a leafhopper abdominal endocuticle protein. Consequently, the altered structural composition of the abdominal endocuticle reduces the overwinter survival of leafhoppers, resulting in a lower incidence of RSMV infection in early-planted rice plants. Our findings illustrate the important roles of RNA interference in virus-vector insect-environment interactions and help explain the annual fluctuations of viral disease epidemics in rice fields.

## INTRODUCTION

Arthropod-borne viruses cause many serious diseases in humans, animals, and plants (1, 2). For example, zika virus, which is transmitted by mosquitoes, has become a public health threat worldwide (3). Rice stripe virus, which is transmitted by planthoppers, poses a serious agricultural threat in rice-growing Asian countries (4). Arthropod-borne viruses typically cause intermittent epidemics (5, 6), for example, Southern rice black-streaked dwarf virus epidemics have exhibited substantial inter-annual dynamics over past decades in Asia (7). However, the mechanism underlying the dynamics of viral disease epidemics remains unknown.

Temperature strongly influences the interactions among insect species (8, 9) and between viruses and their vector insects, with the latter interactions influencing insects’ survival, population dynamics, and geographic distribution (10). Temperature also influences the occurrence and prevalence of insect-borne viruses. For example, the prevalence of most rice viruses is determined mainly by the numbers of viruliferous insect vectors after overwintering (11, 12).

Rice stripe mosaic virus (RSMV) is a member of the genus *Cytorhabdovirus* of the family *Rhabdoviridae*. It was first discovered on rice plants in Southern China in 2015 (13). RSMV is transmitted by the non-migratory *Recilia dorsalis* in a persistent-propagative manner. It cannot be transmitted mechanically, vertically, or in seeds (14). Although *R. dorsalis* nymphs and adults can both transmit RSMV, nymphs have a higher transmission efficiency (14). In most *R. dorsalis* adults, RSMV spreads to all organs by around 12 days after the initial feeding on diseased plants (15). *R. dorsalis* individuals overwinter in the debris of harvested rice plants, as well as in gramineous weeds such as *Digitaria sanguinalis, Monochoria vaginalis,* and *Eleusine indica*. In early spring, overwintered viruliferous *R. dorsalis* individuals transmit the virus to new rice seedlings, causing primary infection and rice stripe mosaic disease (RSMD). Thus, two to three generations of leafhoppers that develop in rice fields become viruliferous, spread the virus, and cause reinfection. After three to four generations, newly hatched nymphs become viruliferous and disperse into other rice fields causing disease outbreaks. Before and after summer rice is harvested, adults *R. dorsalis* migrate to the debris of harvested rice plants or gramineous weeds for overwintering (16). Once present in an area, RSMV spreads rapidly and can cause annual epidemics. However, from 2015 to 2017, the severity of epidemics in the initially affected rice–-rowing areas varied, with the RSMD incidence ranging from 10% to 15%. This incidence decreased to 5% to 8% from 2018 to 2019 but increased to 16%–20% from 2020 to 2021 (16). Notably, the mean temperatures in the fields from November to March are much higher than the temperatures used in this laboratory studies. The occurrence of RSMD can, therefore, show significant annual fluctuations. Overwintering affects not only the survival of individual insect but also the size and geographical distribution of an insect population (17). Thus, the overwinter survival of viruliferous *R. dorsalis* individuals appears to be a key factor determining the geographic distribution and epidemics of RSMV.

RNA interference (RNAi) can serve as a cell-intrinsic means of immunity against viruses (18, 19). In addition to endogenous small RNAs, however, virus-derived small-interfering RNAs (vsiRNAs) are synthesized by host cells and can downregulate host gene expression (20–22). Thus, vsiRNAs can play key roles in virus infection.

In this study, we surveyed the *R. dorsalis* populations in Guangdong Province, China, to determine the overwinter presence of RSMV during four consecutive years. We also studied the effects of RSMV infection on the cold tolerance of *R. dorsalis*. The following three questions were addressed: (i) Does RSMV infection affect the overwinter survival of *R. dorsalis*? (ii) How does RSMV reduce the cold tolerance of *R. dorsalis*? (iii) What is the epidemiological significance of RSMV infection of *R. dorsalis*? We used *R. dorsalis* adults to answer these questions because this species usually overwinters at the adult stage (23). Finally, we explored a mechanism involving the downregulation of *R. dorsalis RdABD-5* transcription by the RMSV-derived small-interfering RNA vsiR-t00355379. These findings expand our knowledge of the impacts of viruses on insect vectors and provide further insights into the ecological relationships between viral pathogens, their vectors, and their environment.

## RESULTS

### RSMV infection decreases the overwinter survival of *R. dorsalis*

**Fig 1:**
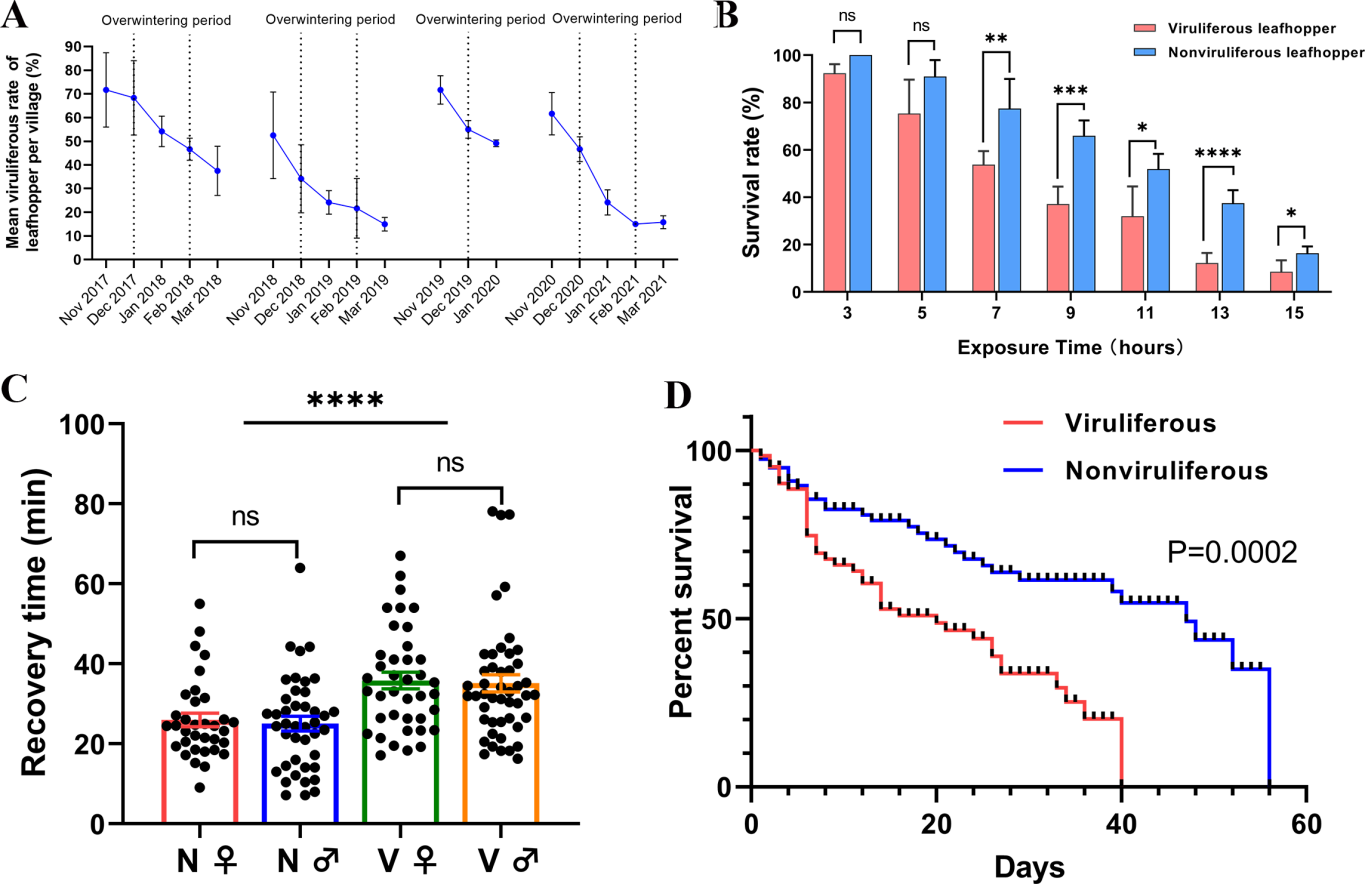
Effects of RSMV on the cold tolerance of *R. dorsalis*. (**A**) The mean viruliferous rate among the *R. dorsalis* populations in four villages of Luoding city, Guangdong Province, China, during the winter seasons of 2017–2021. Graphs show the mean ± SD. (**B**) The survival rates of viruliferous and nonviruliferous leafhoppers after exposure to 0°C for different durations. Each temperature tolerance test was performed in quintuplicate, and each replicate included 15 leafhoppers. Graphs show the mean ± SE. ns, not significant, *P < 0.05, **P < 0.01, ***P < 0.001, ****P < 0.0001, determined using Student’s *t* test. (**C**) Comparison of chill coma recovery time between viruliferous (**V**) and nonviruliferous (**N**) male or female leafhoppers after exposure to 0°C for 7 h. Graphs show the mean ± SE (*n* > 30). ns, not significant, ****P < 0.0001, determined using Student’s *t* test. (**D**) Survival curves of viruliferous and nonviruliferous leafhoppers after exposure to 0°C for 7 h. The chi-square was test used for statistical analyses (*n* = 100).

To evaluate the effects of RSMV on *R. dorsalis* in rice fields, we determined the overwinter viruliferous rates of *R. dorsalis* populations during winter in Luoding city, Guangdong, China, from November 2017 to March 2021. The overwintering *R. dorsalis* individuals were mainly adults, and their population number sharply decreased over winter (Fig. S1A). The viruliferous rate reached a maximum of 71.67% (86/120) before overwintering (November of the current year). After overwintering (February of the following year), however, the viruliferous rate was less than 15% (18/120) (Fig. 1A). These results suggest that viruliferous *R. dorsalis* individuals were more susceptible to death result from cold stress than their nonviruliferous counterparts.

### RSMV infection decreases the cold tolerance of *R. dorsalis*

To test the influence of RSMV on the cold tolerance of *R. dorsalis*, we first used laboratory assays to measure the effects of exposure to cold stress (0°C) on survival. After 7 h exposure to 0°C, viruliferous and nonviruliferous adult *R. dorsalis* differed significantly with respect to survival (Fig. 1B), whereas no significant difference in survival was observed between females and males (Fig. S1B). The time required for 50% of the test insects to succumb to death (LT50, 8.06 h) and LT90 (13.17 h) of viruliferous insects were both significantly shorter than the LT50 (11.27 h) and LT90 (17.61 h) of nonviruliferous insects (Fig. S1C). These results indicate that cold stress is detrimental to the survival of viruliferous *R. dorsalis* and that cold tolerance is unrelated to gender.

The chill coma recovery time (CCRT) is a measure used commonly to assess cold tolerance in cold susceptible insects (24). After exposure to 0°C for 7 h, the CCRT of viruliferous *R. dorsalis* was 35.44 min, compared with 25.47 min for their nonviruliferous counterparts. Females and males did not differ in terms of CCRT (Fig. 1C). We also determined whether brief exposure to cold stress would shorten the longevity of *R. dorsalis*. Viruliferous and nonviruliferous adults were pre-exposed to cold (0°C, 7 h) and subsequently maintained at 25°C to study their longevity. The longevity of viruliferous adults (23.00 d) was significantly shorter than that of nonviruliferous adults (47.00 d) (Fig. 1D). In contrast, viruliferous and nonviruliferous adult leafhoppers did not differ in terms of longevity after pre-exposure to 25°C for 7 h (Fig. S1D). The results indicate that RSMV infection decreased the cold tolerance of *R. dorsalis*, while short duration cold stress shortened the longevity of viruliferous *R. dorsalis*.

### RSMV infection decreases the expression of genes encoding insect cuticular proteins

**Fig 2:**
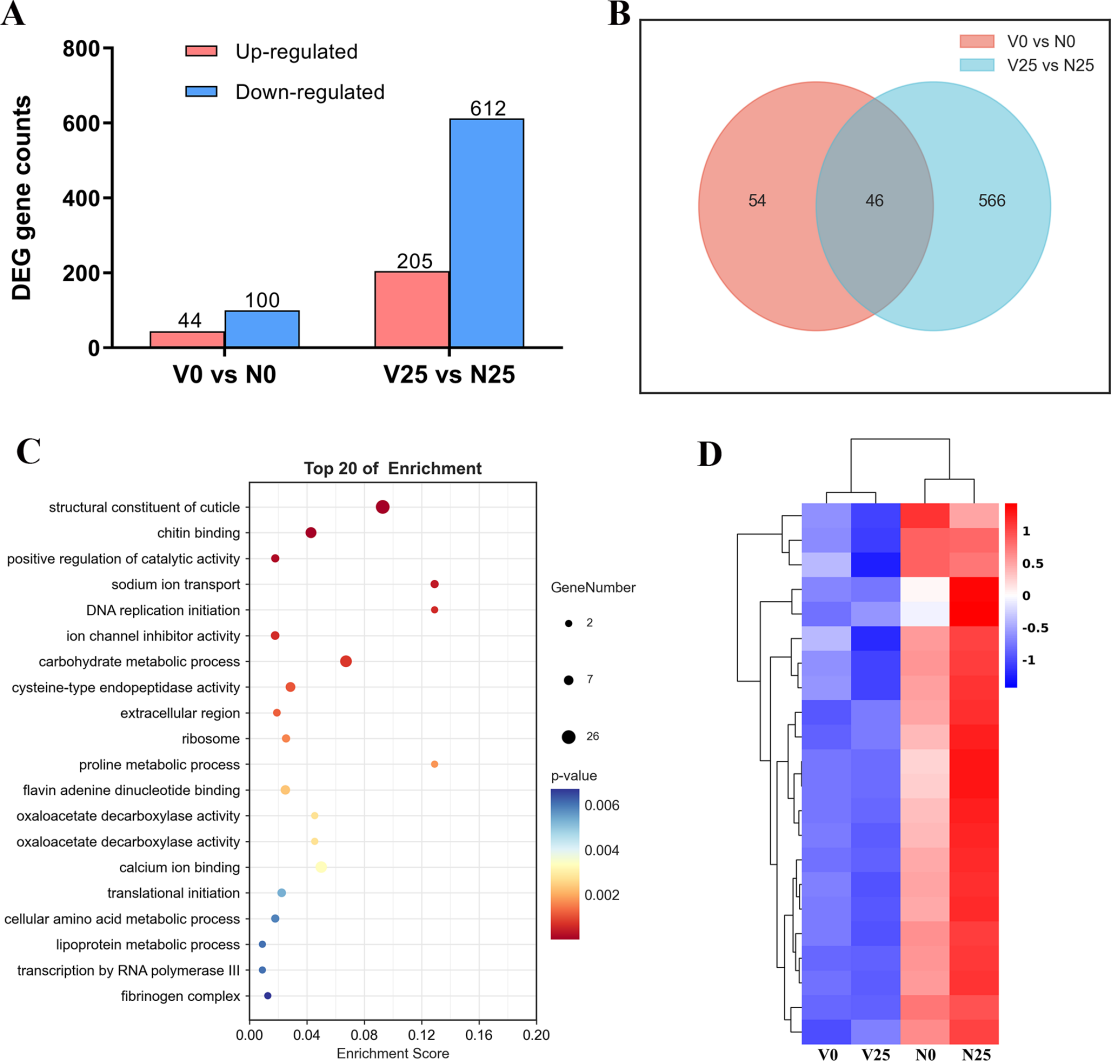
Transcriptomic analyses of viruliferous and nonviruliferous leafhoppers at 0°C and 25°C for 7 h. (**A**) The numbers of regulated unigenes in pairwise comparisons. Each sample name indicates the infection and treatment status: V0 (viruliferous, cold-treated at 0°C), N0 (nonviruliferous, cold-treated at 0°C), V25 (viruliferous, reared at 25°C), and N25 (nonviruliferous, reared at 25°C). The numbers indicate the differentially expressed genes. (**B**) Venn diagram of the numbers of diﬀerentially expressed unigenes induced by RSMV infection in leafhoppers exposed to under 0°C and 25°C (shown as the number of downregulated genes per group when comparing V0 vs N0 and V25 vs **N25**). (**C**) GO analysis of the downregulated genes in leafhoppers in response to RSMV infection and cold stress. The numbers indicate the genes included in each GO category, and the *P*-value indicates data reliability. (**D**) Hierarchical clustering shows the leafhopper CP genes specifically downregulated in response to RSMV infection. The expression levels were calculated according to the log2(fold change) values. Red and blue indicate higher and lower levels of CP gene expression, respectively.

To study the molecular mechanisms associated with the decreased cold tolerance of RSMV-infected *R. dorsalis*, we extracted messenger RNA from viruliferous and nonviruliferous *R. dorsalis* maintained at 0°C or 25°C for 7 h (V0: viruliferous, cold-treated at 0°C for 7 h; N0: nonviruliferous, cold-treated at 0°C for 7 h; V25: viruliferous, reared at 25°C for 7 h; and N25: nonviruliferous, reared at 25°C for 7 h). We constructed transcriptomes for each treatment group using the Illumina sequencing platform and compared them to determine which genes were upregulated or downregulated. RSMV infection upregulated 44 genes and downregulated 100 genes in the V0 group (vs the N0 group) and upregulated 205 genes and downregulated 612 genes in the V25 group (vs the N25 group) (Fig. 2A). To confirm the associations of these dysregulated genes with RSMV infection and cold stress, 20 genes that exhibited the same regulation orientation in reverse-transcription quantitative polymerase chain reaction (RT-qPCR) as revealed in Transcriptomic sequencing were selected, with a few genes without consistent regulation orientation (Table S1). RSMV infection led to more downregulated genes than upregulated genes (Fig. 2A). Forty-six downregulated genes were common to both V0 (vs N0) and V25 (vs N25) (Fig. 2B). Gene Ontology (GO) term enrichment analysis showed that these downregulated genes were mainly enriched in structural constituents of the cuticle pathway (Fig. 2C). Hierarchical clustering analysis showed that genes encoding insect CPs were, indeed, downregulated in viruliferous leafhoppers compared with nonviruliferous leafhoppers (Fig. 2D). Insect CPs are major components of the cuticle and are involved in resistance to environmental stress (25–27). Therefore, we focused on a functional study of CP genes with regard to the effects of RSMV infection on the cold tolerance of *R. dorsalis*.

### RSMV-derived vsiRNAs suppress the transcription of genes encoding CPs in *R. dorsalis*

**Fig 3:**
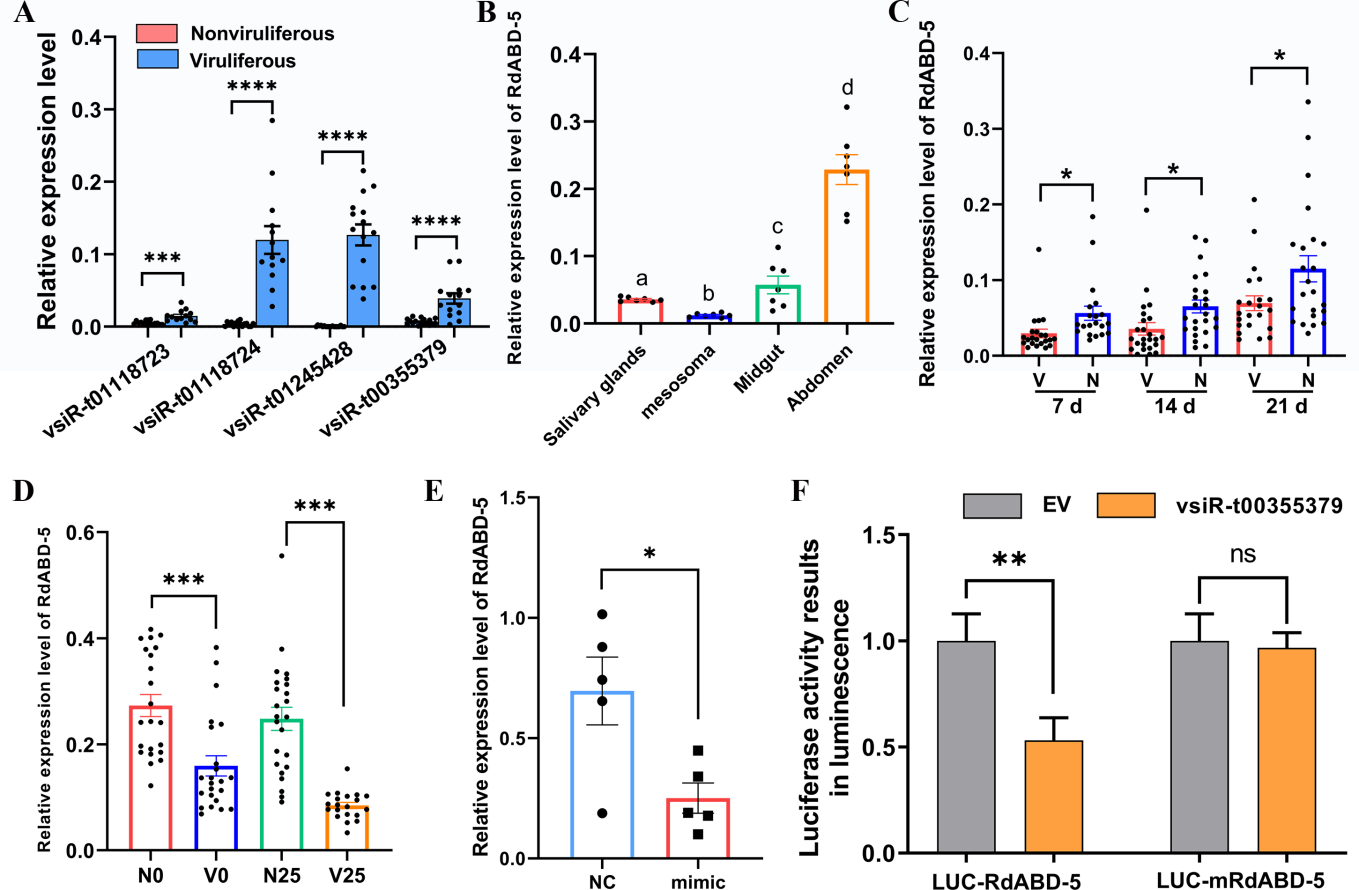
RSMV-derived siRNA represses *R. dorsalis* CP gene transcription. (**A**) The relative expression levels of four vsiRNAs in newly emerged viruliferous (**V**) and nonviruliferous (**N**) leafhopper adults. Graphs show the mean ± SE (*n* > 10). ***P < 0.001, ****P < 0.0001, determined using Student’s *t* test. (**B**) The relative expression levels of *RdABD-5* in nonviruliferous leafhopper adults. Graphs show the mean ± SE (*n* = 7). Different letters represent significant differences determined using Student’s *t* test. (**C**) The relative expression levels of *RdABD-5* in nonviruliferous (**N**) and viruliferous (**V**) leafhopper adults at different days post-acquisition on RSMV-infected plants, every five leafhoppers as a sample. Graphs show the mean ± SE. *P < 0.05, ***P < 0.001, determined using Student’s *t* test. (**D**) Relative expression levels of RdABD-5 in N and V leafhopper adults exposed to 0℃ and 25℃, respectively, for 7 h, every five leafhoppers as a sample. Graphs show the mean ± SE. ***P < 0.001, determined using Student’s *t* test. (**E**) The relative levels of *RdABD-5* mRNA in nonviruliferous leafhoppers at 3 days after injection with the vsiR-t00355379 mimic. Graphs show the mean ± SE. *P < 0.05, determined using Student’s *t* test. (**F**) Detected luciferase activity in *N. benthamiana* leaves. Graphs show the mean luminescence signal counts ± SD per second. ns, not significant, **P < 0.01, determined using Student’s *t* test. LUC-RdABD-5 and LUC-mRdABD-5 indicate the expression vectors incorporating the *RdABD-5* CDS and a CDS with a mutated targeting site, respectively. VsiR-t00355379 and EV represent the expression vector in which the miR528 sequences were replaced by vsiR-t00355379 sequences and the empty vector, respectively.

As more genes were downregulated by RSMV infection than were upregulated, based on the transcriptome, we tested whether vsiRNAs were involved in the downregulation of leafhopper genes. A total of 8,197 RSMV-derived vsiRNAs were identified through small RNA sequencing of viruliferous leafhopper samples. To screen for *R. dorsalis* genes putatively targeted by RSMV-derived vsiRNAs, 8,197 vsiRNAs and 46 genes downregulated in both viruliferous groups of leafhoppers (including five putative CP genes) were selected for target gene prediction. Only gene cluster-12250.48756 matched the highest predicted score and lowest free energy when using psRNATarget and miRanda methods (Tables S2 and S3). Four putative vsiRNAs, namely vsiR-t01118724, vsiR-t00355379, vsiR-t01118723, and vsiR-t01245428, targeted the same site in gene cluster-12250.48756 (Fig. S2A). RT-qPCR was used to detect these four vsiRNAs in viruliferous leafhoppers (Fig. 3A). Sanger sequencing showed that only vsiR-t01245428 and vsiR-t00355379 possessed the correct 21-nucleotide (nt) sequences (Fig. S2B). We chose vsiR-t00355379 for further studies because it was expressed at higher levels in viruliferous leafhoppers than in nonviruliferous leafhoppers (Fig. 3A) and matched gene cluster-12250.48756 with the highest predicted score and lowest free energy (Tables S2 and S3).

*In silico* sequence analysis revealed that gene cluster-12250.48756 produced a putative 103-amino-acids (aa) protein that contained a signal peptide and chitin-binding domain 4 (Fig. S2A). A phylogenetic tree constructed from the CP sequences of other insects revealed that the identified CP clustered with HvABD-5-like, showing the highest identity (79.2%) with *Homalodisca vitripennis* (Fig. S2C). Gene sequence analysis revealed that cluster-12250.48756 has the Rebers and Riddiford consensus sequence (28) and contains a conserved RR-1 domain (Fig. S2D), which belongs to a class of endocuticle structural glycoproteins that are mainly expressed in the insect abdomen. We named this putative protein RdABD-5. *RdABD-5* was found to be highly expressed in the abdomen, followed by the midgut, whereas the lowest expression was found in the mesosoma of nonviruliferous adults (Fig. 3B).

To determine whether *RdABD-5* is regulated by RSMV in adult leafhoppers, its expression was detected at three time points: 7, 14, and 21 days, after virus acquisition. Compared with the expression in nonviruliferous leafhoppers, the expression of *RdABD-5* was downregulated by 15.62%, 45.37%, and 39.60% after exposure to RSMV for 7, 14, and 21 days, respectively (Fig. 3C). At 0°C and 25°C, the expression of *RdABD-5* in viruliferous leafhoppers decreased by 41.63% and 65.85%, respectively, compared with that in nonviruliferous leafhoppers. There was no significant difference in the expression of *RdABD-5* between leafhoppers exposed to 0°C and 25°C (Fig. 3D). In nonviruliferous leafhoppers, the *RdABD-5* transcript level significantly decreased at 3 days after the injection of a synthetic vsiR-t00355379 mimic compared with that in the negative controls (Fig. 3E). The predicted target site of vsiR-t00355379 is from 75 to 96 bp upstream of the *RdABD-5* coding region. The seed region from nt 2 to nt 8 of the vsiRNA perfectly matched the target sequence (Fig. S2A). In *Nicotiana benthamiana* leaves transfected with the *RdABD-5* coding sequence (CDS), the relative luciferase activity was significantly decreased in the presence of vsiR-t00355379, while mutations at the target site corresponding to the seed region of vsiR-t00355379 eliminated the ability of vsiR-t00355379 to suppress luciferase activity (Fig. 3F). These results indicate that *RdABD-5* expression is related to RSMV infection and demonstrate that vsiR-t00355379 suppresses *RdABD-5* transcription but is not affected by temperature changes.

### Silencing *RdABD-5* expression reduces the cold tolerance of *R. dorsali*s

**Fig 4:**
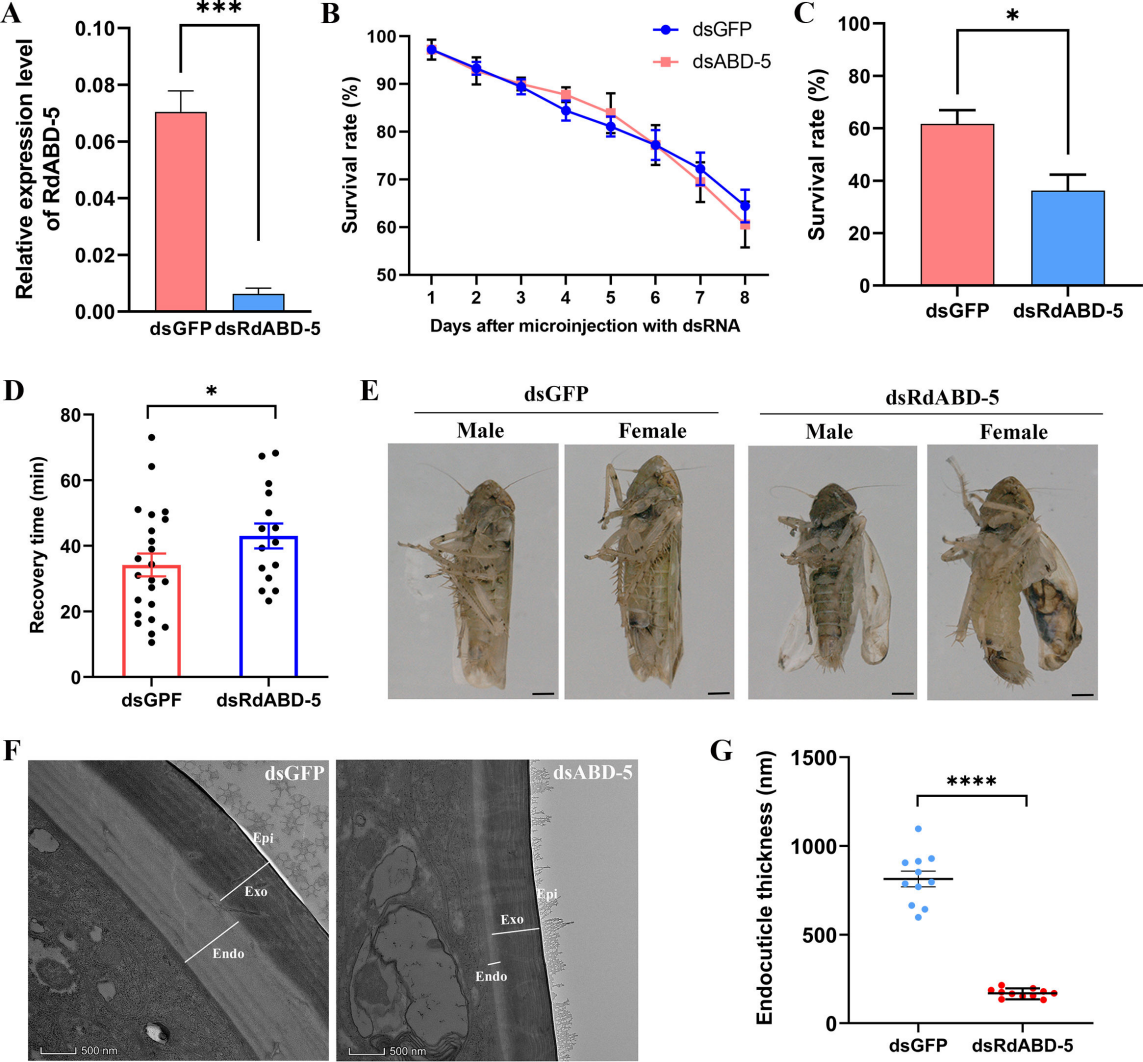
Silencing *RdABD-5* expression reduces cold tolerance in *R. dorsalis*. (**A**) The relative expression of *RdABD-5* at day 5 post-injection with dsRdABD-5 or dsGFP was determined using RT-qPCR. Graphs show the mean ± SE. ***P < 0.001, determined using Student’s *t* test. (**B**) The survival rate of leafhoppers at day 5 post-injection with dsRdABD-5 or dsGFP after exposure to 25°C (*n* = 100). (**C**) The survival rate of leafhoppers injected with dsGFP and dsRdABD-5 after exposure to 0°C for 7 h. Each temperature tolerance test was performed in quintuplicate, and each replicate included 15 leafhoppers. Graphs show the mean ± SE of five independent experiments. *P < 0.05, determined using Student’s *t* test. (**D**) The chill coma recovery time of leafhoppers injected with dsGFP and dsRdABD-5 after exposure to 0°C for 7 h (*n* = 30). Graphs show the mean ± SE, *P < 0.05. (**E**) Phenotypes of male or female leafhoppers at 5 days post-injection with dsRdABD-5 and dsGFP. Scale bars = 200 µm. (**F**) TEM ultrastructural analysis of the leafhopper adult abdominal cuticle at 5 days post-injection with dsRdABD-5 and dsGFP. Epi, epicuticle; Exo, exocuticle; and Endo, endocuticle. Scale bars = 500 nm. (**G**) TEM analysis of the abdominal endocuticle thickness of leafhoppers injected with dsGFP and dsRdABD-5 (*n* = 11). ****P < 0.0001, determined using Student’s *t* test.

As *RdABD-5* expression was repressed by RSMV infection, we tested the functional role of RdABD-5 in insect cold stress tolerance. First, double-stranded RNAs (dsRNA) targeting *RdABD-5* (dsRdABD-5) and GFP (dsGFP, control) were synthesized *in vitro* and injected into the hemocoel (between the second and third abdominal segments) of newly emerged adults. The efficiency of RNAi was analyzed using RT-qPCR at 5 days post-injection. The expression of *RdABD-5* in the newly emerged adults injected with dsRdBAD-5 was downregulated by 91.12% compared with the control (Fig. 4A). The survival rate of dsRdABD-5-injected insects was not significantly different from that of the controls at 25°C (Fig. 4B). To verify the effects of RdABD-5 on the cold tolerance of leafhoppers, dsRdABD-5-injected leafhoppers were kept at 0°C for 7 h. The corresponding survival rate of dsRdABD-5-injected *R. dorsalis* was 36.22%, significantly lower than the 61.66% survival rate of dsGFP-injected *R. dorsalis* (Fig. 4C). The CCRT of *R. dorsalis* after *RdABD-5* silencing was 41.51 min, significantly longer than that of the control group (30.27 min) (Fig. 4D). Additionally, when *RdABD-5* was silenced in *R. dorsalis*, the abdominal phenotype was unchanged, but the wings were irregularly curled or appeared deformed compared with the wings of dsGFP-injected controls (Fig. 4E). However, compared with dsGFP-injected *R. dorsalis*, the endocuticle lamellar structure in dsRdABD-5-injected *R. dorsalis* was reduced and thinner, with unclear boundaries (Fig. 4F and G). The abdominal phenotype of viruliferous leafhoppers was similar to that of nonviruliferous leafhoppers (Fig. S3A). However, the cuticular structure of the abdomen was significantly thinner in viruliferous leafhoppers than in nonviruliferous leafhoppers (Fig. S3B and C). In addition, in viruliferous leafhoppers, the lamellar structure of the endocuticle was reduced, the delineation between lamellae was not clear, and the endocuticle was loose (Fig. S3B and C). These results demonstrate that RdABD-5 plays a functional role in the cold stress tolerance of *R. dorsalis*. The downregulation of *RdABD-5* led to reduced cold tolerance in *R. dorsalis* by reducing the thickness of its abdominal endocuticle.

### Monitoring the incidence rate of RSMV in Guangdong Province, China

To determine the epidemiological role of RSMV in reducing the cold tolerance of *R. dorsalis* in rice fields, we surveyed the RSMV incidence rate during the early-planted rice and late-planted rice periods for four consecutive years in Guangdong Province, China. The RSMV incidence rate in early-planted rice plants was significantly lower than that in late-planted rice plants (Fig. S4). These results show a positive correlation between the reduced overwinter survival of viruliferous *R. dorsalis* and the lower incidence of RSMV in early-planted rice.

## DISCUSSION

Under cold winter temperatures, the acquisition of RSMV from infected plants by nonviruliferous *R. dorsalis* is blocked, and RSMV currently cannot be transmitted vertically (14). Without an effective supplementary virus source, such as RSMV-infected rice plants, overwintering viruliferous *R. dorsalis* is the only source of virus for future transmission. Our field study revealed the reduced overwinter survival of RSMV-infected *R. dorsalis* in rice fields (Fig. 1A). Laboratory experiments demonstrated that RSMV infection decreased the cold tolerance of *R. dorsalis* (Fig. 1B through D). These results, together with data showing a lower incidence rate of RSMV in early-planted (vs late planted) rice plants (Fig. S4) indicate that RSMV infection reduces the survival of *R. dorsalis* over winter. This interaction has helped reduce the severity of RSMV epidemics and explains the observed variation in the frequency of RSMV epidemics in Guangdong Province, China over the past 5 years.

Transcriptomic analysis indicated that the expression of genes encoding CPs, which are important for cuticle structural integrity in insects (29–31), was downregulated in viruliferous leafhoppers compared with nonviruliferous leafhoppers (Fig. 3). CPs are involved in insects’ responses to temperature stress (32–35), suggesting that they play a key role in the survival of leafhoppers under cold conditions. Small RNA sequencing revealed that the downregulation of insect CP genes targeted by RSMV-derived small-interfering RNAs reduced the formation of lamellae and led to a thinner lamellar structure in the endocuticle in RSMV-infected leafhoppers (Fig. 4F and G; Fig. S3), and these effects correspond to a decrease in cold tolerance. These findings highlight the important role of vsiRNAs in virus–vector–environment interactions.

To facilitate efficient virus transmission, plant viruses have adapted to achieve persistent infection and maintenance in vectors. For example, barley yellow dwarf virus-PAV increases the surface temperature of an infected host plant and enhances the thermal tolerance of its aphid vector, *Rhopalosiphum padi*. This increased tolerance allows *R. padi* to occupy the higher and warmer regions of infected host plants when it is displaced from cooler regions by competition with the larger aphid species *R. maidis*. This effect has led to an expansion of the fundamental niche of the vector (36). However, some viruses may have adverse effects on the survival of their insect vector. For example, Southern rice black-streaked dwarf virus increases the death rate of its vector, the planthopper *Sogatella furcifera*, under extreme cold stress (37). In addition, rice gall dwarf virus reduces the post-winter survival of its vector, the leafhopper *R. dorsalis* (38). However, the molecular mechanism underlying these effects is not clear. The piRNA-guided pre-mRNA splicing of oocyte development-related genes, which is a conserved mechanism in animals, that regulates density-dependent reproductive strategy (39). In the current study, the evidence shows that RSMV reduces the cold tolerance of its vector and, thus, does not promote the dispersal of viruliferous leafhoppers or virus transmission by these vectors from year to year. Therefore, in response to RSMV infection, vector leafhoppers limit their population size via endocuticle thickness reduction mediated by vsiRNA. This reduces the vector populations that is exposed to the cold temperatures of winter.

Our data show lower expression of *RdABD-5* in viruliferous leafhoppers in warm weather than in cold weather (Fig. 3D). This suggests that insects have a thinner abdominal endocuticle during warmer seasons, leading to better survival of viruliferous leafhoppers and a higher incidence rate of RSMV in rice fields in warm weather than in cold weather. Our results in Fig. S4 support this suggestion: namely, the RSMV incidence in late-planted rice plants under warm weather was significantly higher than that in early-planted rice under cold weather. Furthermore, cold weather stress weakened the immunity of the leafhoppers, causing them to be more susceptible to RSMV infection and related death. Altering the endocuticle thickness may be only one of the factors responsible for reduced cold tolerance. We cannot rule out potential effects of other abdominal endocuticle family proteins, either alone or in combination, or of unknown mechanisms that may modulate insect cold tolerance, including the osmoregulatory capacity and physiological processes (40, 41). Therefore, leafhoppers exposed to the combination of RSMV infection and cold stress are more likely than uninfected leafhoppers to die, thus reducing the future incidence of RSMV infection after a high incidence in the previous year.

**Fig 5:**
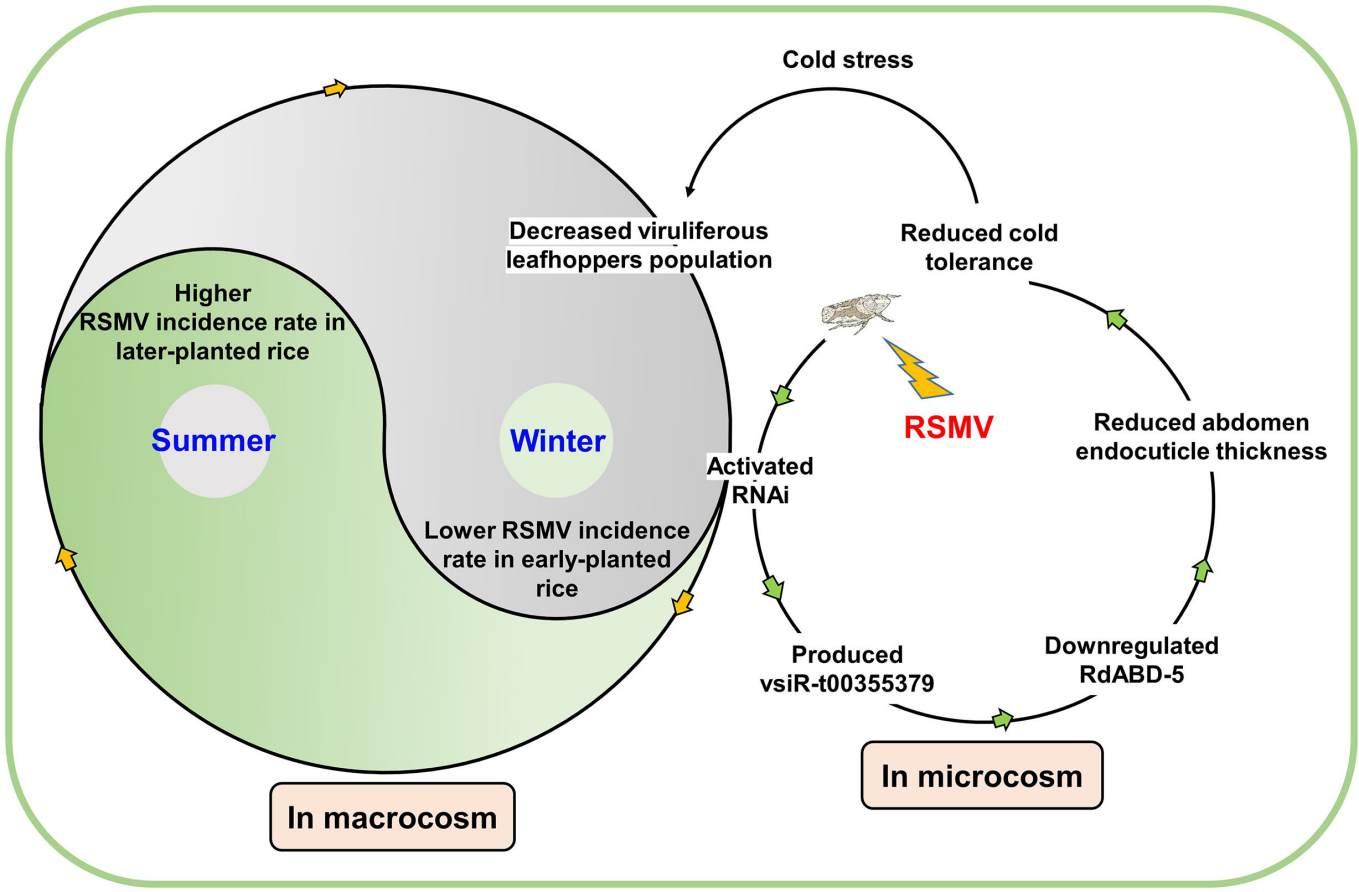
A model of intermittent RSMV epidemics. In macrocosm, because leafhopper *R. dorsalis* cannot migrate over long distances, overwintering plays an important role in virus transmission among hosts between seasons. Cold stress decreases the survival of viruliferous leafhoppers in winter, which, in turn, reduces the incidence of RSMD in the early-planted rice period. After the accumulation of viruliferous leafhoppers, a large number of viruliferous leafhoppers transfer to late-planted rice plants, leading to more severe disease among these plants and accessing the next winter. In microcosm, RSMV infection activates the RNAi pathway in leafhoppers, leading to the production of many virus-derived small interfering RNAs. In particular, vsiR-t00355379 was found to downregulate the expression of *RdABD-5*, which encodes an abdominal endocuticle structural glycoprotein, thus altering of the vector’s abdominal endocuticle structure. This change ultimately reduces the vector’s cold tolerance in winter and reduces the chance of an RSMV epidemic in the following year.

In conclusion, we identified a positive correlation between infection with the plant virus RSMV and reductions in the cold tolerance and overwinter survival of its vector *R. dorsalis*. This situation has created a bottleneck of viral persistence in rice fields (Fig. 5). Our findings revealed one mechanism that contributes, at least in part, if not entirely, to the reduced cold tolerance of *R. dorsalis* via RSMV-mediated repression of *RdABD-5* expression. Epidemiologically, lowering the overwinter survival of viruliferous *R. dorsalis* after RSMV infection reduces the incidence rate of RSMV in early-planted rice fields and, thus, attenuates the incidence of RSMD epidemics in the following year. The combined effect of viral pathogens and temperature in terms of regulating the survival of multicellular viral host provides insights into the development of approaches to reduce viral epidemics by targeting vsiRNA-mediated mechanisms.

## MATERIALS AND METHODS

### Field monitoring of viruliferous *R. dorsalis* and investigation of RSMV incidence rate in Guangdong Province, China

To investigate the effects of RSMV on *R. dorsalis* over winter, we investigated variations in the field population and viruliferous rate of leafhoppers in Luoding city, Guangdong Province, China, from November 2017 to March 2021. This area included four villages (Taiping, Chuanbu, Luoping, and Luojing). To investigate the field populations, *R. dorsalis* individuals were randomly collected from six rice fields in each village after repelling with a sweeping net using a chessboard sampling method (23). To determine the viruliferous rate, 120 leafhoppers were randomly collected from 6 rice fields in each village to ensure a consistent sample size. During each survey, adjacent survey sampling times were separated by intervals of approximately about 30 days. To monitor the incidence of RSMV infection in rice fields across the 4 villages, 100 plants were randomly collected from 6 rice fields in each village per year during both the early (May to July) and late (August to October) rice planting periods from 2018 to 2021. RSMV was detected in the collected rice plants using an RT-PCR method (13).

### Rice plants, virus materials, and insect vectors

The rice plants (*Oryza sativa* L. cv. “Nipponbare”) used in this study were maintained in our laboratory. Plant culture and virus inoculation were performed according to previously published procedures (14). Briefly, the rice seeds were germinated by soaking in warm water (37°C for 2 days), sown in a covered plastic box containing soil–matrix (soil:matrix = 1:1) and maintained in insect-proof net cages at 25°C ± 1°C and 75% ± 5% relative humidity under a 16:8 h (L:D) photoperiod. The seedlings were inoculated with viruliferous fourth-instar nymphs propagated from RSMV-infected rice plants maintained in our laboratory. Confirmed RSMV-infected and RSMV-uninfected plants were used to propagate of *R. dorsalis*. The leafhoppers were maintained in insect-proof net cages.

### Cold tolerance tests for *R. dorsalis*

As adult leafhoppers are mainly found in winter fields (23), we used newly emerged male and female leafhoppers in cold tolerance tests. The temperature of villages in Luoding city range from 0°C to 4°C during winter, we used an extreme temperature (0°C) for the cold tolerance test, and a moderate temperature of 25°C as the control. To assess cold tolerance, the newly emerged adult leafhoppers were exposed directly to 0°C for 3, 5, 7, 9, 11, 13, and 15 h in a programmable cold bath. Each temperature tolerance test was conducted in quintuplicate, and each replicate included 15 insects. After cold exposure, the leafhoppers were transferred immediately to three-leaf-stage rice seedlings at 25°C for 3 h. The vitality and mortality of leafhoppers were determined by lightly touching them with a brush pen. Individuals that moved were scored as alive, and those that failed to move were scored as dead. After the experiments were completed, all insects were individually tested using RT-PCR to confirm RSMV infection. The survival rates of the viruliferous and nonviruliferous groups were calculated for each treatment. Linear regression was used to estimate the LT50 or LT90 based on the above data. To test the longevity of insects after the cold tolerance test, the viruliferous and nonviruliferous newly emerged male and female leafhoppers exposure to 0°C for 7 h were subsequently transferred to a 25°C environment for recovery. After 3 h, the leafhoppers were separated and housed individually in plastic tubes containing three-leaf rice seedlings. Their survival status was monitored daily. For the CCRT experiments, newly emerged male and female adult leafhoppers were transferred to tubes and exposed to 0°C for 7 h in a programmable cold bath. After cold treatment, the insects were transferred immediately to clean filter paper at 25°C for 3 h and monitored to determine the recovery time. The recovery status of individual insects after cold treatment was determined by touching them with a brush pen every 30 min.

### Transcriptomic and small RNA sequencing

Transcriptomic sequencing was conducted as previously described (42). Four groups were tested in triplicate: V0 (viruliferous, cold-treated at 0°C for 7 h), N0 (nonviruliferous, cold-treated at 0°C for 7 h), V25 (viruliferous, reared at 25°C for 7 h), and N25 (nonviruliferous, reared at 25°C for 7 h). Each test comprised 60 newly emerged adults. Each group sample was flash-frozen in liquid nitrogen immediately after exposure to 0°C or 25°C for 7 h and then sent to Novogene (Beijing, China) for transcriptomic sequencing. Clean reads were used for *de novo* assembly with Trinity (43) using the default parameters and contigs were assembled into unigene sequences. The unigenes were subjected to NT, NR, Swiss-Prot, KEGG, and COG annotation, with the *E*-value threshold set at 10^−5^.

For small RNA sequencing, we sent viruliferous leafhoppers (reared at 25°C) to Novogene. Each analysis was performed in triplicate, and each replicate included 50 newly emerged adults. Total RNA was extracted using a TRIzol reagent kit (Invitrogen, Carlsbad, CA, USA), and RNA molecules in the size range of 18–30 nt were enriched by polyacrylamide gel electrophoresis. Next, 3′ adapters were added and 36–48 nt RNAs were enriched. Subsequently, 5′ adapters were ligated to the RNAs. The ligation products were reverse-transcribed and amplified by PCR. The 140–160 bp PCR products were enriched to generate a cDNA library and sequenced using the Illumina HiSeq Xten platform. Further filtering of the raw reads from the adapter sequences was performed using in-house Perl scripts. Then, filtered reads in the range of 18–25 nt were aligned with RSMV genome sequences (GenBank ID: KX525586.2) using bowtie software (http://bowtie-bio.sourceforge.net) with two mismatches. The obtained reads were termed as RSMV-derived small interfering RNAs and used for further analysis.

### Bioinformatics, phylogenetic analysis and prediction of vsiRNAs binding sites

Nucleotide sequence analysis and unigene assembly were performed using Vector NTI (44). Open reading frames (ORFs) were predicted using the ORF finder tool on the National Center for Biotechnology Information (NCBI) website (http://www.ncbi.nlm.nih.gov/gorf/gorf.html). The nt and aa sequences were aligned with those of other insect CP genes using the Clustal W program (45). The conserved domains in the aa sequences were predicted using the Conserved Domain Database tool on the NCBI website (https://www.ncbi.nlm.nih.gov/Structure/cdd/wrpsb.cgi) and the online InterPro tool (http://www.ebi.ac.uk/interpro/). A phylogenetic tree was constructed from the deduced aa sequences in MEGA 11, using the neighbor-joining method with a bootstrap of 1,000 replicates (46). The GenBank accession numbers of the CP sequences used for comparison and phylogenetic analysis are given in Table S4. To analyze the predicted vsiRNA-binding sites, all vsiRNAs were subjected to target gene prediction using psRNATarget (47) with a cutoff value of 3 and miRanda (48) with a cutoff value of −160 and a free energy threshold of −30 kcal mol^−1^.

### Double-stranded RNA synthesis and delivery

*RdABD-5* was amplified by PCR and used to synthesize a 325 bp dsRNA targeting the *RdABD-5* coding region (dsRdABD-5), using the T7 RiboMAX Express RNAi System kit (Promega, Madison, WI, USA) according to the manufacturer’s protocol. A 420 bp dsRNA targeting the gene encoding GFP (dsGFP) was amplified as a negative control. The dsRNA fragments were purified using sodium acetate and ethanol precipitation. Subsequently, A dsRNAs were administered to newly emerged adult leafhoppers leafhopperss in a volume of 27.5 nl (0.5 µg/µL) by microinjection with a Nanoject II Auto-Nanoliter Injector (Spring), and the insects were raised on RSMV-free rice seedlings. All primers used in this study are listed in Table S5.

### Injection of vsiR-t00355379 mimic

The vsiR-t00355379 mimic and negative control (NC) sequences were chemically synthesized from the double-stranded vsiR-t00355379 (Table S8) and NC sequences (RiboBio, Guangzhou, China). Subsequently, 27.5 nl of the vsiR-t00355379 mimic or NC (100 µM) was administered to nonviruliferous newly emerged adult leafhoppers by microinjection using a Nanoliter 2000 system (World Precision Instruments). The leafhoppers were collected for RNA isolation at 3 d post-injection.

### RNA isolation and quantitative RT-PCR

Total RNA was extracted from the whole bodies of five adult leafhoppers, as well as from 10 salivary glands, 10 mesosomas, 10 guts, and 10 abdomens using RNA isolate Extraction Reagent (Vazyme, Nanjing, China) as previously described (14). Next, cDNA was synthesized from total RNA using oligo (dT) primers and reverse transcriptase (Takara, China). The PCR procedure was as follows: one cycle at 95°C for 30 s, followed by 40 cycles at 95°C for 5 s and 60°C for 30 s. Quantitative PCR analyses were performed using a CFX96 Touch real-time PCR detection system (Bio-Rad, Hercules, CA, USA) and SYBR Premix Ex Taq II (Takara, China). Each experiment was performed in triplicate, and three technical RT-qPCR replicates were performed per biological repeat. Real-time quantification of the vsiRNAs was performed using stem-loop RT-qPCR as previously described (49), and the expression levels of vsiRNAs were normalized to leafhopper *Actin*. The primers are listed in Table S5.

### vsiRNA target validation

To test the vsiRNA targeting leafhopper *RdABD-5*, a vsiRNA expression vector was constructed using the miR528-OE vector skeleton (50). The miR528 sequences were replaced by vsiRNA sequences using overlapping PCR. We used amiRNA-F and amiRNA-R primers to amplify osa-miR528, which was then subjected to a second round of PCR amplification using one of the following primer pairs: amiRNA-F and vsiR-t00355379-II, vsiR-t00355379-I and vsiR-t00355379-IV, or vsiR-t00355379-III and amiRNA-R. The resulting PCR products were mixed and used in a third round of PCR amplification with the amiRNA-F and amiRNA-R primers, and the product was cloned into the *Sal*I and *Hind*III restriction enzyme sites of the vector to generate Actin-P: vsiR-t00355379. To construct the *RdABD-5* reporter vector, the *RdABD-5* CDS was amplified using the Luc-RdABD5-F and Luc-RdABD5-R primers and cloned into the *Sac*I site to generate 35S: LUC-RdABD-5. A CDS with a mutated targeting site was cloned into the same site to generate 35S: LUC-mRdABD-5. The miR528-OE vector plasmid was used as a control. All of the vectors were individually transformed into *Agrobacterium tumefaciens* strain C58C1, which was cultured to an optical density at 600 nm of 1.0 and then used to infiltrate *N. benthamiana* leaves. The infiltrated leaves were sprayed with 1 mM luciferin (Promega), and luciferase activity was measured after 72 h using a low-light cooled charge-coupled device imaging apparatus (LB 985 NightSHADE system, Berthold Technologies, Germany). The primers used in this experiment are listed in Table S5.

### Transmission electron microscopy

For ultrastructural analysis, transmission electron microscopy (TEM) was performed using a previously described method (14). Briefly, fourth-instar nymphs reared on RSMV-infected rice plants for 10 days and adults aged 5 days after dsRNA injection were collected. The abdominal cuticles were dissected and fixed in 4% glutaraldehyde (100 mM phosphate buffer, pH 7.0), follow by 1% osmium tetroxide overnight. The fixed tissues were dehydrated in an alcohol gradient series, excised on an ultra-microtome and embedded in Spurr’s resin containing 1% uranyl acetate and lead citrate for staining. The stained sections were collected on copper grids and observed using a ThermoFisher Talos L120C transmission electron microscope.

### Statistical analysis

All data were analyzed using GraphPad Prism 8.0 software (GraphPad Software Inc.). the error bars represent the standard deviation or standard error. Between-group data comparisons were performed using Student’s *t* test. Survivor curves were analyzed using the chi-square test.

## Data Availability

We have deposited the RNA-seq data in the NCBI Sequence Read Archive (Accession No. PRJNA985382). The GenBank accession number of RdABD-5 is OR161370.

## SUPPLEMENTAL MATERIAL

Fig. S1 mbio.03211-23-s0001.tif Effects of RSMV infection on the cold tolerance of *R. dorsalis*.

Fig. S2 mbio.03211-23-s0002.tif Bioinformatics analysis of the *R. dorsalis* cuticular protein RdABD-5.

Fig. S3 mbio.03211-23-s0003.tif Analysis of the cuticles of viruliferous and nonviruliferous leafhoppers.

Fig. S4 mbio.03211-23-s0004.tif Incidence rate of RSMV during the early-planted rice and late-planted rice periods in four villages in Luoding city, Guangdong Province, China, from 2018 to 2021.

Supplemental Material mbio.03211-23-s0005.docx Fig. S1 to S4 legends; Tables S1 to S5.

ASM does not own the copyrights to Supplemental Material that may be linked to, or accessed through, an article. The authors have granted ASM a non-exclusive, world-wide license to publish the Supplemental Material files. Please contact the corresponding author directly for reuse.
