# Supplementary figures and images for: Reduced cold tolerance of viral-infected leafhoppers attenuates viral persistent epidemics
Source: mBio. 2024 Apr 2;15(5):e03211-23. doi: 10.1128/mbio.03211-23 (PMC11077983; doi:10.1128/mbio.03211-23)

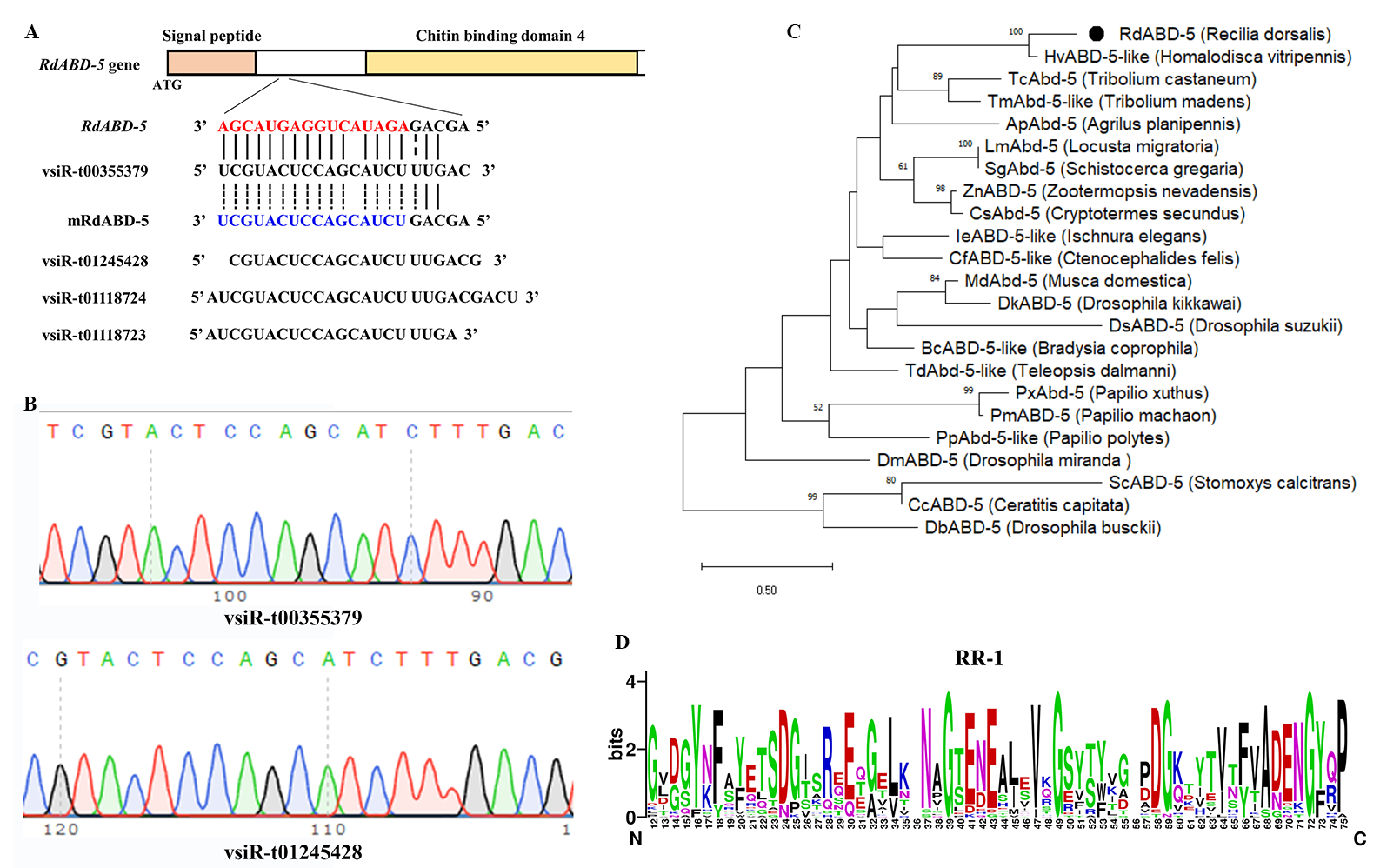

Supplement: Fig. S2 — Bioinformatics analysis of the R. dorsalis cuticular protein RdABD-5. [file mbio.03211-23-s0002.tif]
