# Supplementary material for: Reduced cold tolerance of viral-infected leafhoppers attenuates viral persistent epidemics
Source: mBio. 2024 Apr 2;15(5):e03211-23. doi: 10.1128/mbio.03211-23 (PMC11077983; doi:10.1128/mbio.03211-23)
Supplement: Supplemental Material — Fig. S1 to S4 legends; Tables S1 to S5. [file mbio.03211-23-s0005.docx]

**Supplemental material figure legends**

**FIG S1** Effects of RSMV infection on the cold tolerance of *R. dorsalis*. (A) The mean *R. dorsalis* population numbers in four villages in Luoding, Guangdong province, China, during the winter seasons from 2017 to 2021. Graphs show the mean ± SD. (B) The survival rates of viruliferous (V) and nonviruliferous (N) male or female leafhoppers after exposure to 0°C for 7 h. Each temperature tolerance test was performed in quintuplicate, and each replicate included 15 leafhoppers; the data are presented as the mean ± SE. ns, not significant, * P < 0.05, ** P < 0.01, determined using Student’s t test. (C) Linear fit of the relative survival rates of viruliferous and nonviruliferous leafhoppers after exposure to 0°C for 7 h. (D) Life curves of viruliferous and nonviruliferous leafhoppers after exposure to 25℃ for 7 h. The chi-squared test was used for statistical analyses (n = 100).

**FIG S2** Bioinformatics analysis of the *R. dorsalis* cuticular protein RdABD-5. (A) Sequence alignment of four vsiRNAs with their predicted target sites in the CDS of *RdABD-5*. The alignment sequences are shown in red. Mutated nucleotides in the target (mRdABD-5) are shown in blue. (B) Sanger sequencing showed that vsiR-t01245428 and vsiR-t00355379 possessed the correct 21-nucleotide (nt) sequences. (C) Phylogenetic analysis of insect ABD-5 based on its amino acid sequence. The phylogenetic tree was constructed using the neighbor-joining method with 1000 bootstrap replicates. The GenBank accession numbers are listed in Table S4. (D) RR-1 motif analysis of homologous ABD-5 sequences from different species, created using the online Weblogo tool (https://weblogo.threeplusone.com/).

**FIG S3** Analysis of the cuticles of viruliferous and nonviruliferous leafhoppers. (A) Phenotypes of viruliferous and nonviruliferous male and female leafhoppers. Scale bars = 200 μm. (B) TEM ultrastructural analysis of the abdominal cuticles of viruliferous and nonviruliferous newly emerged adult leafhoppers. Epi: epicuticle; Exo: exocuticle; Endo: endocuticle. Scale bars = 500 nm. (C) TEM analysis of the abdominal endocuticle thickness of viruliferous and nonviruliferous leafhoppers (n = 11). **P < 0.01, determined using Student’s t test.

**FIG S4** Incidence rate of RSMV during the early-planted rice and late-planted rice periods in four villages in Luoding city, Guangdong Province, China, from 2018 to 2021.

| **Table S1. Validation of the RNA-seq results using RT-qPCR.** | | | | | |
| --- | --- | --- | --- | --- | --- |
| **No.** | **Gene ID** | **V0 vs N0** | | **V25 vs N25** | |
|  |  | **RNA-seq** | **RT-qPCR** | **RNA-seq** | **RT-qPCR** |
| 1 | Cluster-12250.48337 | 1.88 | -0.23 | -0.22 | -0.77 |
| 2 | Cluster-12250.55238 | 1.56 | 3.59 | 0.47 | 0.55 |
| 3 | Cluster-12250.45812 | 1.47 | 1.15 | 0.46 | -0.66 |
| 4 | Cluster-12250.62940 | 1.23 | 3.57 | -0.68 | -0.178 |
| 5 | Cluster-12250.58849 | 1.16 | 0.63 | -0.97 | -2.19 |
| 6 | Cluster-12250.53049 | -1.01 | -1.76 | -0.45 | -1.61 |
| 7 | Cluster-12250.23775 | -1.11 | -3.44 | -0.78 | -1.11 |
| 8 | Cluster-12250.16701 | -2.16 | -2.53 | -4.99 | -3.65 |
| 9 | Cluster-12250.32905 | -2.49 | -3.08 | -1.79 | -2.69 |
| 10 | Cluster-12250.53243 | 2.16 | 4.52 | -0.66 | 5.77 |
| 11 | Cluster-12250.58093 | 1.17 | 2.37 | -0.03 | 1.19 |
| 12 | Cluster-12250.32556 | -0.16 | 0.22 | -1.23 | -3.55 |
| 13 | Cluster-12250.64802 | -1.05 | -2.43 | -1.38 | -1.47 |
| 14 | Cluster-12250.16701 | -0.34 | -2.33 | -4.99 | -3.1 |
| 15 | Cluster-12250.34421 | -0.19 | 0.14 | -1.43 | 0.71 |
| 16 | Cluster-12250.48332 | -1.32 | -1.45 | -1.6 | -1.98 |
| 17 | Cluster-12250.38875 | -0.41 | 0.77 | -1.04 | 0.45 |
| 18 | Cluster-12250.23039 | 0.52 | -3.21 | -5.34 | -4.36 |
| 19 | Cluster-12250.22143 | -1.91 | -2.55 | -2.27 | -3.61 |
| 20 | Cluster-12250.51007 | -1.07 | -2.2.1 | -1.42 | -1.95 |

**Table S2. Prediction of 63 putative pairs of vsiRNA targets using psRNATarget.**

| **vsiRNA ID** | **Target** | **Expectation** | **vsiRNA_start** | **vsiRNA_end** | **Target_start** | **Target_end** | **vsiRNA_aligned_fragment** | **alignment** | **Target_aligned_fragment** |
| --- | --- | --- | --- | --- | --- | --- | --- | --- | --- |
| vsiR-t00355379 | Cluster-12250.48756 | 1.5 | 1 | 21 | 66 | 86 | UCGUACUCCAGCAUCUUUGAC | ::.:::: ::::::::::: | AGCAGAGAUACUGGAGUACGA |
| vsiR-t01118723 | Cluster-12250.48756 | 2.5 | 1 | 21 | 67 | 87 | AUCGUACUCCAGCAUCUUUGA | ::.:::: ::::::::::: | GCAGAGAUACUGGAGUACGAG |
| vsiR-t01245428 | Cluster-12250.48756 | 2.5 | 1 | 21 | 65 | 85 | CGUACUCCAGCAUCUUUGACG | ::.:::: :::::::::: | CAGCAGAGAUACUGGAGUACG |
| vsiR-t00535455 | Cluster-12250.54079 | 2 | 1 | 21 | 2054 | 2074 | AGAGGGGAAGAAGAAGUUUGG | :.::.:::::::::::.::: | CUAAGCUUCUUCUUCCUCUCG |
| vsiR-t00539814 | Cluster-12250.80260 | 2.5 | 1 | 21 | 327 | 347 | AGUACUUGGAGUCUUUAACCU | :: ::.:::::::::::: | GUGUCAAGGACUCCAAGUACA |
| vsiR-t00143495 | Cluster-12250.70665 | 2.5 | 1 | 21 | 428 | 447 | UAUAAUGAAUUAGUUGGCAAC | : ::::::::. ::::::::: | GAUGCCAACUG-UUCAUUAUA |
| vsiR-t00784466 | Cluster-12250.60665 | 2.5 | 1 | 21 | 1 | 21 | CUUCUUGACCGAUUACCUGCC | :::::: ::::::::::::. | GGCAGGAAAUCGGUCAAGAGC |
| vsiR-t00775381 | Cluster-12250.60615 | 2.5 | 1 | 24 | 1408 | 1431 | CUACUAUGGGGUGUCCCCACAACC | :::: :.:.::::.:::::: | UAGGGUGGAGGCGCCCCGUAGUAG |
| vsiR-t00894167 | Cluster-12250.60615 | 2.5 | 1 | 21 | 972 | 992 | UGGAGUUAGAAUUUCAAGAAA | ::.:::: ::: :::::::: | CUUUUUGAUAUUAUAACUCCA |
| vsiR-t00239170 | Cluster-12250.56653 | 2.5 | 1 | 21 | 34 | 54 | UUGAAUAAGUCAGUAUGUGAC | :::::::.: :.:::::.:: | AUCACAUAUUUAUUUAUUUAA |
| vsiR-t01642301 | Cluster-12250.56653 | 2.5 | 1 | 20 | 35 | 54 | UUGAAUAAGUCAGUAUGUGA | :::::::.: :.:::::.:: | UCACAUAUUUAUUUAUUUAA |
| vsiR-t00831483 | Cluster-12250.49763 | 2.5 | 1 | 21 | 381 | 401 | UACCCACCUCAACAUCCAGCC | : :: :.:: ::::::::::: | GACUCGGUGCUGAGGUGGGUA |
| vsiR-t00191076 | Cluster-12250.35388 | 2.5 | 1 | 20 | 188 | 207 | UGUUGACUUUAAAGAACACU | .:::::.:::::: :.:::: | GGUGUUUUUUAAAUUUAACA |
| vsiR-t00071919 | Cluster-12250.28354 | 2.5 | 1 | 21 | 1840 | 1860 | AAAAGAACUCAGUCAUAUCCU | : :::::::.:::::::: | CCAAGAUGACUGGGUUCUUUU |
| vsiR-t00176481 | Cluster-12250.28354 | 2.5 | 1 | 21 | 1841 | 1861 | UAAAAGAACUCAGUCAUAUCC | : :::::::.:::::::: | CAAGAUGACUGGGUUCUUUUC |
| vsiR-t01455180 | Cluster-12250.28354 | 2.5 | 1 | 21 | 1764 | 1784 | UCAAUCCCUCCAGACCAAGAA | :::: ::::: :::::::. | GCCUUGUUCUGGUGGGAUUGG |
| vsiR-t00309632 | Cluster-12250.27469 | 2.5 | 1 | 21 | 710 | 730 | UAAUACACUACACGAGAUUGU | :::.:: ::::::::.::. | GAAAUUUCCUGUAGUGUGUUG |
| vsiR-t00532979 | Cluster-12250.63464 | 3 | 1 | 21 | 403 | 423 | ACUGUCUGCUUCUGGAGCCGG | :::: :::: ::.:::.:: | GAGGCUACAGAUGCGGACGGU |
| vsiR-t00557273 | Cluster-12250.63464 | 3 | 1 | 21 | 404 | 424 | CACUGUCUGCUUCUGGAGCCG | :::: :::: ::.:::.::: | AGGCUACAGAUGCGGACGGUG |
| vsiR-t01178151 | Cluster-12250.63464 | 3 | 1 | 22 | 403 | 424 | CACUGUCUGCUUCUGGAGCCGG | :::: :::: ::.:::.::: | GAGGCUACAGAUGCGGACGGUG |
| vsiR-t00509735 | Cluster-12250.60665 | 3 | 1 | 21 | 709 | 729 | UUUCCCUCUUUCAAUAGACCU | .:::::::: . ::.:::::: | GGGUCUAUUCGCAGGGGGAAA |
| vsiR-t00574907 | Cluster-12250.60665 | 3 | 1 | 21 | 230 | 250 | CUGGCUCCCUGUCAGCCUGAC | ::::.::::: :::::: : | UGCAGGUUGACACGGAGCCUG |
| vsiR-t01270209 | Cluster-12250.60665 | 3 | 1 | 22 | 229 | 250 | CUGGCUCCCUGUCAGCCUGACC | ::::.::::: :::::: : | AUGCAGGUUGACACGGAGCCUG |
| vsiR-t01511617 | Cluster-12250.60665 | 3 | 1 | 21 | 285 | 305 | UCUGUUACCCUGAUCAUGGAG | : ::::::::: ::.::.:: | CCCCAUGAUCACAGUGACGGA |
| vsiR-t01511618 | Cluster-12250.60665 | 3 | 1 | 22 | 284 | 305 | UCUGUUACCCUGAUCAUGGAGA | : ::::::::: ::.::.:: | CCCCCAUGAUCACAGUGACGGA |
| vsiR-t00795306 | Cluster-12250.60615 | 3 | 1 | 21 | 1875 | 1895 | GAGUUAGAAUUUCAAGAAAAC | :::.: ::: ::::.:::: | CAUUUUUGGAAUUUCUGACUC |
| vsiR-t01357018 | Cluster-12250.58084 | 3 | 1 | 21 | 342 | 362 | GUCGUACUGGCAGCCCCAUGA | : : :::: :::::::::: | AAAGGAGGCUACCAGUACGAC |
| vsiR-t00293281 | Cluster-12250.58013 | 3 | 1 | 21 | 1081 | 1101 | ACCGUCAUGAUCUCCUGCUUC | :. :::::::.:.:::..::: | GGUGCAGGAGGUUAUGGUGGU |
| vsiR-t00689844 | Cluster-12250.58013 | 3 | 1 | 22 | 1080 | 1101 | ACCGUCAUGAUCUCCUGCUUCA | :. :::::::.:.:::..::: | AGGUGCAGGAGGUUAUGGUGGU |
| vsiR-t00320561 | Cluster-12250.57732 | 3 | 1 | 21 | 495 | 515 | UUCUUACCUGACUUGGUCCUC | : ::::..:: :.::::.::: | GUGGACUGAGGCGGGUAGGAA |
| vsiR-t00576262 | Cluster-12250.57732 | 3 | 1 | 21 | 496 | 516 | CUUCUUACCUGACUUGGUCCU | ::::..:: :.::::.:::: | UGGACUGAGGCGGGUAGGAAG |
| vsiR-t01559945 | Cluster-12250.56653 | 3 | 1 | 22 | 356 | 377 | UGGAAGAGAUUAAGACCGCUGU | ::::::: : ::::::::: | CGAGCGGUCGUCAUCUCUUCCC |
| vsiR-t00170489 | Cluster-12250.54211 | 3 | 1 | 21 | 287 | 307 | AAACAAAUUAAGGCUGUUAGC | : :. ::.::::::::::: | UAUUAUUGCUUUAAUUUGUUU |
| vsiR-t00380792 | Cluster-12250.54079 | 3 | 1 | 21 | 2495 | 2515 | AUCUUGAUCUCGUUUGAGUCC | : ::::::: :: ::..:::: | GCACUCAAAAGAAAUUGAGAU |
| vsiR-t00765958 | Cluster-12250.54079 | 3 | 1 | 21 | 2085 | 2105 | CGAGGAAGGUAAGGGAAGGAG | ::::.:.: .:::::.::: | AACCUUUCUUGGCCUUCUUCG |
| vsiR-t01057336 | Cluster-12250.54079 | 3 | 1 | 21 | 96 | 116 | AGAAGUCAAGACAGCUAAGGC | : ::::: :::::::::: | GAAUAAGCUGGCUUGACUUCU |
| vsiR-t01157052 | Cluster-12250.54079 | 3 | 1 | 20 | 2161 | 2180 | CAAAGAAGGCUGAGAAGGCU | :::: .:. :::::::::: | CGCCUGUUUCGCCUUCUUUG |
| vsiR-t01664982 | Cluster-12250.53315 | 3 | 1 | 21 | 570 | 590 | UUGUUAUCUUAAGAUCCUUGC | ..: ::::::.:::::::: | UUGAAAAUCUUAGGAUAACAA |
| vsiR-t00722065 | Cluster-12250.51007 | 3 | 1 | 21 | 435 | 455 | AUCCUCUUCGUCAUCCAGGAA | :::::::.::: ::.::::: | UUCCUGGGUGAGGAGGAGGAG |
| vsiR-t00181871 | Cluster-12250.44367 | 3 | 1 | 21 | 176 | 196 | AAUUGAUGACGGAUCCCUUGG | .:: :::::: ::::::::: | UCAUGGGAUCAAUCAUCAAUU |
| vsiR-t00733569 | Cluster-12250.44367 | 3 | 1 | 21 | 175 | 195 | AUUGAUGACGGAUCCCUUGGA | .:: :::::: :::::::: | AUCAUGGGAUCAAUCAUCAAU |
| vsiR-t00189618 | Cluster-12250.41616 | 3 | 1 | 21 | 489 | 509 | UCGGAUGGAGAACCUUGCAAU | ::.:. :::: :::::::: | UCUGUAGAGUUCACCAUCCGA |
| vsiR-t00867515 | Cluster-12250.41616 | 3 | 1 | 22 | 488 | 509 | UCGGAUGGAGAACCUUGCAAUC | ::.:. :::: :::::::: | CUCUGUAGAGUUCACCAUCCGA |
| vsiR-t00146415 | Cluster-12250.41168 | 3 | 1 | 21 | 712 | 732 | AAUUAGUUGGCAACCUGUACU | .:: :::: :::::.::.::: | GGUUCAGGGUGCCAGCUGAUU |
| vsiR-t00334639 | Cluster-12250.41168 | 3 | 1 | 21 | 711 | 731 | AUUAGUUGGCAACCUGUACUU | :.:: :::: :::::.::.:: | AGGUUCAGGGUGCCAGCUGAU |
| vsiR-t00466485 | Cluster-12250.41168 | 3 | 1 | 21 | 713 | 733 | GAAUUAGUUGGCAACCUGUAC | :: :::: :::::.::.:::: | GUUCAGGGUGCCAGCUGAUUC |
| vsiR-t00492675 | Cluster-12250.41168 | 3 | 1 | 21 | 714 | 734 | UGAAUUAGUUGGCAACCUGUA | : :::: :::::.::.:::: | UUCAGGGUGCCAGCUGAUUCC |
| vsiR-t00579027 | Cluster-12250.41168 | 3 | 1 | 19 | 715 | 733 | GAAUUAGUUGGCAACCUGU | :::: :::::.::.:::: | UCAGGGUGCCAGCUGAUUC |
| vsiR-t00706456 | Cluster-12250.41168 | 3 | 1 | 21 | 547 | 567 | AGGAAAGAAGAUUCAAGAGAC | .:.:::..:.::::::.:: | UAUUUUUGGGUUUUCUUUUCU |
| vsiR-t00878794 | Cluster-12250.41168 | 3 | 1 | 22 | 713 | 734 | UGAAUUAGUUGGCAACCUGUAC | :: :::: :::::.::.:::: | GUUCAGGGUGCCAGCUGAUUCC |
| vsiR-t01007118 | Cluster-12250.41168 | 3 | 1 | 19 | 714 | 732 | AAUUAGUUGGCAACCUGUA | : :::: :::::.::.::: | UUCAGGGUGCCAGCUGAUU |
| vsiR-t00082534 | Cluster-12250.35388 | 3 | 1 | 21 | 1410 | 1430 | AUCAUGGAUUCAGAGACCACU | :::::: : :::.:.:::: | UAUGGUCUAUAAAUUCGUGAU |
| vsiR-t00121307 | Cluster-12250.35388 | 3 | 1 | 21 | 1022 | 1042 | UGGAGGGAGAAACAGUUGAUC | ::..::: :::.::::::.: | AAUUGACUAUUUUUCCCUCUA |
| vsiR-t01505095 | Cluster-12250.35388 | 3 | 1 | 21 | 664 | 684 | UCUCCUCCGAUGAUUCUGGUA | ..::::: . :::::::::: | GGUCAGAAGUUUCGGAGGAGA |
| vsiR-t01563521 | Cluster-12250.35388 | 3 | 1 | 20 | 1023 | 1042 | UGGAGGGAGAAACAGUUGAU | ::..::: :::.::::::.: | AUUGACUAUUUUUCCCUCUA |
| vsiR-t00267745 | Cluster-12250.30935 | 3 | 1 | 21 | 182 | 202 | AUAAGGACUAUUGCGUUGUCU | :::.:: .:::::::::: | CUCCAAUGCCGUAGUCCUUAU |
| vsiR-t00678201 | Cluster-12250.30935 | 3 | 1 | 21 | 661 | 681 | AAUGACAGAAUCUCCUCCUCA | ::::: :.:::::::.:: | UCUGGAGGUGGUUCUGUCGUU |
| vsiR-t01002504 | Cluster-12250.30935 | 3 | 1 | 19 | 663 | 681 | AAUGACAGAAUCUCCUCCU | ::::: :.:::::::.:: | UGGAGGUGGUUCUGUCGUU |
| vsiR-t01291519 | Cluster-12250.30935 | 3 | 1 | 20 | 663 | 682 | GAAUGACAGAAUCUCCUCCU | ::::: :.:::::::.:: | UGGAGGUGGUUCUGUCGUUG |
| vsiR-t00370092 | Cluster-12250.28354 | 3 | 1 | 21 | 352 | 372 | AAGGAGAAGUUCCCCUGUGCU | :: :::::::::: :.::: | CCCAAAGGGGAACUUGUUCUU |
| vsiR-t01361952 | Cluster-12250.27469 | 3 | 1 | 19 | 667 | 685 | GUGGCCUCAAUGUCGGUAU | ::..:::.::::.::..:: | AUGUCGAUAUUGGGGUUAC |
| vsiR-t01286673 | Cluster-12250.21175 | 3 | 1 | 21 | 352 | 372 | GAACAUGUCCAGUGUGAUCAU | :::::..::::. :::::: | CCGAUCAUGCUGGGGAUGUUC |
| vsiR-t00725832 | Cluster-12250.14919 | 3 | 1 | 22 | 392 | 413 | AUGAUACCCGGGAGGUCCUCAC | : :::::::::.: :: ::::: | GGGAGGACCUCUCCGGCAUCAU |

**Table S3. Prediction of 33 putative pairs of vsiRNA targets using miRanda.**

| **vsiRNA ID** | **target** | **target_start** | **target_end** | **energy(kcal/mol)** | **score** | **target_region(3'->5')** | **vsiRNA-target_pairing(5'->3')** | **miRNA_sequence(5'->3')** |
| --- | --- | --- | --- | --- | --- | --- | --- | --- |
| vsiR-t01118724 | Cluster-12250.48756 | 32 | 57 | -31.69 | 188 | gAGCATGAGGTCATAGAGACgacctc | x\|\|\|\|\|\|\|\|\|\|\|x\|\|\|\|:\|\|xxxxxx | aTCGTACTCCAGCATCTTTGacgact |
| vsiR-t00355379 | Cluster-12250.48756 | 36 | 56 | -32.28 | 183 | aGCATGAGGTCATAGAGACga | x\|\|\|\|\|\|\|\|\|\|x\|\|\|\|:\|\|xx | tCGTACTCCAGCATCTTTGac |
| vsiR-t01118723 | Cluster-12250.48756 | 37 | 57 | -31.69 | 183 | gAGCATGAGGTCATAGAGAcg | x\|\|\|\|\|\|\|\|\|\|\|x\|\|\|\|:\|xx | aTCGTACTCCAGCATCTTTga |
| vsiR-t00927590 | Cluster-12250.30935 | 400 | 421 | -33.01 | 178 | gACCTGGGAAGACAACGACGag | x\|\|\|\|\|\|\|\|\|\|\|x\|x\|\|\|\|\|xx | tTGGACCCTTCT-TAGCTGCtt |
| vsiR-t01561509 | Cluster-12250.30935 | 399 | 420 | -32.6 | 178 | aCCTGGGAAGACAACGACGAga | x\|\|\|\|\|\|\|\|\|\|x\|x\|\|\|\|\|\|xx | tGGACCCTTCT-TAGCTGCTta |
| vsiR-t01245428 | Cluster-12250.48756 | 35 | 55 | -30.89 | 178 | gCATGAGGTCATAGAGACgac | x\|\|\|\|\|\|\|\|\|x\|\|\|\|:\|\|xxx | cGTACTCCAGCATCTTTGacg |
| vsiR-t00273793 | Cluster-12250.14919 | 307 | 332 | -30.62 | 177 | aAGGACTCTATGAACCCGTCGTGTgg | x\|\|\|\|\|\|\|\|x\|:x\|\|\|xx\|\|\|\|\|\|xx | cTCCTGAGACATGTGG--AGCACAcc |
| vsiR-t00402378 | Cluster-12250.48477 | 470 | 490 | -30.15 | 175 | gTGTCGACCGACACATGTCga | x\|\|\|\|\|\|\|\|\|\|\|xx\|:\|\|\|xx | tACAGCTGGCTGACTGCAGga |
| vsiR-t00235580 | Cluster-12250.32905 | 462 | 482 | -33.42 | 173 | tGCCCCTACGTCCGCTTgaac | x\|\|\|\|\|\|\|\|\|\|\|\|x\|\|:xxxx | tCGGGGATGCAGGGGAGacag |
| vsiR-t01270588 | Cluster-12250.58084 | 229 | 250 | -31.51 | 173 | aACCCGGAGGGACTCGTCGcat | x\|\|\|\|\|\|\|\|:x\|\|:\|\|\|\|\|xxx | cTGGGCCTCT-TGGGCAGCaat |
| vsiR-t00889112 | Cluster-12250.32905 | 32 | 52 | -31.79 | 172 | cCGGGAGAAGGAGGACcctcg | x\|\|\|\|\|\|\|\|\|\|\|\|\|x\|xxxxx | tGCCCTCTTCCTCCGGatact |
| vsiR-t01580667 | Cluster-12250.58084 | 614 | 634 | -34.59 | 171 | gCATCGGAGGAGGAGGAGGag | x\|\|\|\|\|\|\|:\|\|\|xx\|\|\|:\|xx | tGTAGCCTTCTCAGCCTTCtt |
| vsiR-t00097633 | Cluster-12250.43986 | 20 | 40 | -31.94 | 171 | gCCGAGGGAATATCGGACTca | x\|\|\|\|\|\|\|\|xxx\|\|\|\|\|\|\|xx | tGGCTCCCTGTCAGCCTGAcc |
| vsiR-t01357018 | Cluster-12250.58084 | 253 | 273 | -31.09 | 171 | cAGCATGACCATCGGAGGAaa | x\|\|\|\|\|\|\|\|\|x\|\|\|\|x\|x\|xx | gTCGTACTGGCAGCCCCATga |
| vsiR-t01505095 | Cluster-12250.35388 | 664 | 684 | -30.13 | 171 | aGAGGAGGCTTTGAAGACTgg | x\|\|\|\|\|\|\|\|\|x:x\|\|\|\|\|:xx | tCTCCTCCGATGATTCTGGta |
| vsiR-t01575818 | Cluster-12250.21175 | 72 | 93 | -34 | 170 | cCCAGCGGCTGGCATGGGTAcc | x\|\|\|\|\|\|\|\|\|\|:\|x:::\|\|\|xx | tGGTCGCCGACTG-GTTCATgt |
| vsiR-t01304279 | Cluster-12250.27469 | 453 | 472 | -33.29 | 170 | cTCCGACCCGACGAGGTGgg | x\|\|\|\|\|\|\|\|x\|x\|\|\|\|:\|xx | gAGGCTGGGATTCTCCGCta |
| vsiR-t00190449 | Cluster-12250.41616 | 437 | 459 | -32.11 | 170 | gCGGAACACTGCCTGGGTCTTga | x\|\|\|\|\|\|\|\|\|\|xx\|:\|\|:\|:\|xx | tGCCTTGTGAC--ATCCGGGAtt |
| vsiR-t01260303 | Cluster-12250.31612 | 29 | 52 | -31.25 | 170 | aAGGCAGACGTAAGAGGAG-GGAca | x\|\|\|\|\|\|\|\|xx\|\|\|\|:\|\|:x\|\|\|xx | cTCCGTCTG--TTCTTCTTACCTga |
| vsiR-t00436494 | Cluster-12250.35388 | 1296 | 1315 | -33.42 | 169 | tGGACACACCGA-ACGGCCag | x\|\|\|\|\|\|\|\|\|\|xx\|\|:\|\|\|xx | aCCTGTGTGGCAATGTCGGaa |
| vsiR-t01241576 | Cluster-12250.60665 | 566 | 585 | -31.12 | 169 | gCCGAACC-CTACAGGAGGcc | x\|\|\|\|\|\|\|x\|\|\|\|\|\|\|x\|:xx | cGGCTTGGAGATGTCCACTtt |
| vsiR-t01223211 | Cluster-12250.54079 | 94 | 118 | -30.51 | 167 | gGAAGAAGCGGAACCTCCGAAGGag | x\|\|\|\|\|\|\|xx\|\|x\|\|\|\|x:\|\|\|\|xx | cCTTCTTCCACT-GGAG-TTTCCac |
| vsiR-t00817908 | Cluster-12250.14919 | 21 | 42 | -30.47 | 167 | cAAGCCTGGAGGGTCTCagcag | x\|\|\|\|\|\|\|:\|\|\|x\|\|\|\|xxxxx | gTTCGGACTTCC-AGAGatatt |
| vsiR-t01060125 | Cluster-12250.80260 | 347 | 368 | -34.41 | 166 | aCTGCACGGTGACCACGACCcc | x\|\|\|\|\|\|\|:\|xx\|\|\|x\|\|\|\|xx | aGACGTGCTAGGGGT-CTGGgg |
| vsiR-t01190116 | Cluster-12250.18580 | 277 | 298 | -31.08 | 166 | gTATCGGAGGTCGCGGAGGAaa | x\|\|\|\|\|\|\|:\|\|\|x\|x\|x\|\|\|xx | cATAGCCTTCAGAGGC-CCTct |
| vsiR-t00354148 | Cluster-12250.56866 | 367 | 389 | -30.14 | 166 | gGGGTCATCGGTACCGGAGGTat | x\|\|\|\|\|\|\|\|:\|x\|xx\|:\|\|\|\|xx | tCCCAGTAGTCTT--CTTCCAtc |
| vsiR-t01061876 | Cluster-12250.48764 | 436 | 455 | -31.39 | 165 | tCTCTCTGCCCCCactcagc | x\|\|\|\|\|\|\|\|\|\|\|\|xxxxxxx | aGAGAGACGGGGGatttgaa |
| vsiR-t01061877 | Cluster-12250.48764 | 435 | 455 | -31.39 | 165 | tCTCTCTGCCCCCactcagcc | x\|\|\|\|\|\|\|\|\|\|\|\|xxxxxxxx | aGAGAGACGGGGGatttgaaa |
| vsiR-t01117986 | Cluster-12250.53275 | 63 | 85 | -30.82 | 165 | gAGCCTACTTGGGGCAGTGccgc | x\|\|\|\|\|\|\|:\|\|x:\|\|x\|\|\|xxxx | aTCGGATGGACATCG-CACatgg |
| vsiR-t00345331 | Cluster-12250.56744 | 954 | 975 | -34.91 | 163 | gGGCTCCGATCGGAGGCGGaga | x\|\|\|\|\|\|\|\|:\|xx\|:\|x\|\|xxx | gCCGAGGCTGGGATTCTCCgct |
| vsiR-t01322131 | Cluster-12250.56744 | 955 | 975 | -34.28 | 163 | gGGCTCCGATCGGAGGCGGag | x\|\|\|\|\|\|\|\|:\|xx\|:\|x\|\|xx | gCCGAGGCTGGGATTCTCCgc |
| vsiR-t00112613 | Cluster-12250.56866 | 148 | 166 | -32.23 | 163 | gAGGCCTAGGG-GT-AGGAga | x\|\|\|\|\|\|\|:\|\|x\|\|x\|\|\|\|xx | tTCCGGATTCCACACTCCTct |
| vsiR-t01114632 | Cluster-12250.30935 | 256 | 276 | -30.3 | 160 | gAGGGGGCGAGGGAGGagcgg | x\|\|\|\|\|\|\|x\|\|::\|\|:xxxxx | aTCCCCCGATCTTTCTcagtc |

| **Table S4. Species and GenBank accession number for the phylogenetic tree used in this study.** | | | |
| --- | --- | --- | --- |
| **Order** | **Species** | **Gene name** | **GenBank accession number** |
| *Hemiptera* | *Recilia dorsalis* | RdABD-5 | OR161370 |
|  | *Homalodisca vitripennis* | HvABD-5-like | XP_046666629.1 |
| *Orthoptera* | *Locusta migratoria* | LmAbd-5 | KX503039 |
|  | *Schistocerca gregaria* | SgAbd-5 | P56561.1 |
| *Blattaria* | *Zootermopsis nevadensis* | ZnABD-5 | KDR18706.1 |
|  | *Cryptotermes secundus* | CsAbd-5 | XP_023704720.1 |
| *Lepidoptera* | *Papilio xuthus* | PxAbd-5 | KPI97917.1 |
|  | *Papilio machaon* | PmABD-5 | KPJ11363.1 |
|  | *Papilio polytes* | PpAbd-5-like | XP_013141199.1 |
| *Diptera* | *Drosophila miranda* | DmABD-5 | XP_017134543.1 |
|  | *Stomoxys calcitrans* | ScABD-5 | XP_013109108.1 |
|  | *Ceratitis capitata* | CcABD-5 | XP_012154723.1 |
|  | *Drosophila busckii* | DbABD-5 | XP_017843899.1 |
|  | *Musca domestica* | MdAbd-5 | XP_005189842.2 |
|  | *Drosophila suzukii* | DsABD-5 | XP_016934998.1 |
|  | *Teleopsis dalmanni* | TdAbd-5-like | XP_037939264.1 |
|  | *Bradysia coprophila* | BcABD-5-like | XP_037044862.1 |
|  | *Drosophila kikkawai* | DkABD-5 | KAH8342514.1 |
| *Coleoptera* | *Tribolium madens* | TmAbd-5-like | XP_044267485.1 |
|  | *Tribolium castaneum* | TcAbd-5 | XP_970596.1 |
|  | *Agrilus planipennis* | ApAbd-5 | XP_018320474.1 |
| *Odonata* | *Ischnura elegans* | IeABD-5-like | XP_046393065.1 |
| *Siphonaptera* | *Ctenocephalides felis* | CfABD-5-like | XP_026467056.1 |

| **Table S5. Sequences of the primers and mimics used in the study.** | |
| --- | --- |
| **Sequence name** | **Sequence (5′ to 3′)** |
| **primers for qPCR** | |
| Actin-F | CGTTCTGGACTCTGGTGATGG |
| Actin-R | CTCAGCAGTGGTTGTGAAGGA |
| RdABD5-qF | CTTCACAACTGATGACGGCAAGA |
| RdABD5-qR | AGCGGCAGGTGGAGGTTATG |
| vsiR-t00355379-RT | GTCGTATCCAGTGCAGGGTCCGAGGTATTCGCACTGGATACGTCAAA |
| vsiR-t00355379-F | CCAGCGTG-TCGTACTCCAGCATC |
| vsiR-t01118723-RT | GTCGTATCCAGTGCAGGGTCCGAGGTATTCGCACTGGATACGACTCAAAG |
| vsiR-t01118723-F | CCAGCGTGATCGTACTCCAGCAT |
| vsiR-t01118724-RT | GTCGTATCCAGTGCAGGGTCCGAGGTATTCGCACTGGATACGACAGTCGT |
| vsiR-t01118724-F | CCAGCGTGATCGTACTCCAGCATCTTTG |
| vsiR-t01245428-RT | GTCGTATCCAGTGCAGGGTCCGAGGTATTCGCACTGGATACGACCGTCAA |
| vsiR-t01245428-F | CCAGCGTGCGTACTCCAGCATCT |
| vsiR-R | CAGTGCAGGGTCCGAGGTAT |
| **Primers for RNAi** | |
| GFP-T7-F | TAATACGACTCACTATAGGGAAGCAGCACGACTTCTTCAAG |
| GFP-T7-R | TAATACGACTCACTATAGGGCGAACTCCAGCAGGACCAT |
| RdABD5-T7-F | TAATACGACTCACTATAGGGGCAGAGATACTGGAGTACGAGAA |
| RdABD5-T7-R | TAATACGACTCACTATAGGGCGGCAGGTGGAGGTTATGAC |
| **Primer for RSMV detection** |  |
| RSMV-L-F | CTCCAACTATCATCCGCTATGC |
| RSMV-L-R | CCATCCGAGATAAGGTCACTGT |
| **Primer for expression plasmid construction** |  |
| amiRNA-F | CGGGGATCCTCTAGAGTCGACCTGCAGCAGCCACA |
| amiRNA-R | AAACGACGGCCAGTGCCAAGCTTCAATCAGTAAATTGAACGGA |
| vsiR-t00355379-I | AGTCGTACTCCAGCATCTTTGACCAGGAGATTCAGTTTGA |
| vsiR-t00355379-II | TGGTCAAAGATGCTGGAGTACGACTGCTGCTGCTACAGCC |
| vsiR-t00355379-III | CTGTCAATGATCCTGGAGTACGATTCCTGCTGCTAGGCTG |
| vsiR-t00355379-IV | AATCGTACTCCAGGATCATTGACAGAGAGGCAAAAGTGAA |
| Luc-RdABD5-F | GGCGGAAAGATCGCCGTGATGCTGACTAGGCTGGTTT |
| Luc-RdABD-R | GCCAAATGTTTGAACGATCTTATGACGTGGTGACGACTG |
| Luc-mRdABD5-F | AGCAGTCTACGACCTCATGCTGAACAACAACATCGGAGTCG |
| Luc-mRdABD5-R | AGCATGAGGTCGTAGACTGCTGGAGGTCGGGCACCCTGGG |
| **Sequences synthesized for injection** |  |
| vsiR-t00355379 mimic | TCGTACTCCAGCATCTTTGAC (sence) |
|  | GTCAAAGATGCTGGAGTACGA (antisence) |
| NC mimic | RiboBio, Guangzhou, China |
